# Supplementary material for: High-quality ultra-fast total scattering and pair distribution function data using an X-ray free-electron laser
Source: IUCrJ. 2025 Jul 22;12(Pt 5):531–47. doi: 10.1107/S205225252500538X (PMC12403175; doi:10.1107/S205225252500538X)
Supplement: Supplementary file 1 [file m-12-00531-sup1.pdf]

IUCrJ

Volume 12 (2025)

Supporting information for article:

High-quality ultra-fast total scattering and pair distribution function data using an X-ray free-electron laser

Adam F. Sapnik, Philip A. Chater, Dean S. Keeble, John S. O. Evans, Federica Bertolotti, Antonietta Guagliardi, Lise J. Støckler, Elodie A. Harbourne, Anders B. Borup, Rebecca S. Silberg, Adrien Descamps, Clemens Prescher, Benjamin D. Klee, Axel Phelipeau, Imran Ullah, Kárel G. Medina, Tobias A. Bird, Viktoria Kaznelson, William Lynn, Andrew L. Goodwin, Bo B. Iversen, Celine Crepisson, Emil S. Bozin, Kirsten M. Ø. Jensen, Emma E. McBride, Reinhard B. Neder, Ian Robinson, Justin Wark, Michal Andrzejewski, Ulrike Boesenberg, Erik Brambrink, Carolina Camarda, Valerio Cerantola, Sebastian Goede, Hauke Höppner, Oliver S. Humphries, Zuzana Konopkova, Naresh Kujala, Thomas Michelat, Motoaki Nakatsutsumi, Alexander Pelka, Thomas R. Preston, Lisa Randolph, Michael Roeper, Andreas Schmidt, Cornelius Strohm, Minxue Tang, Peter Talkovski, Ulf Zastrau, Karen Appel and David A. Keen

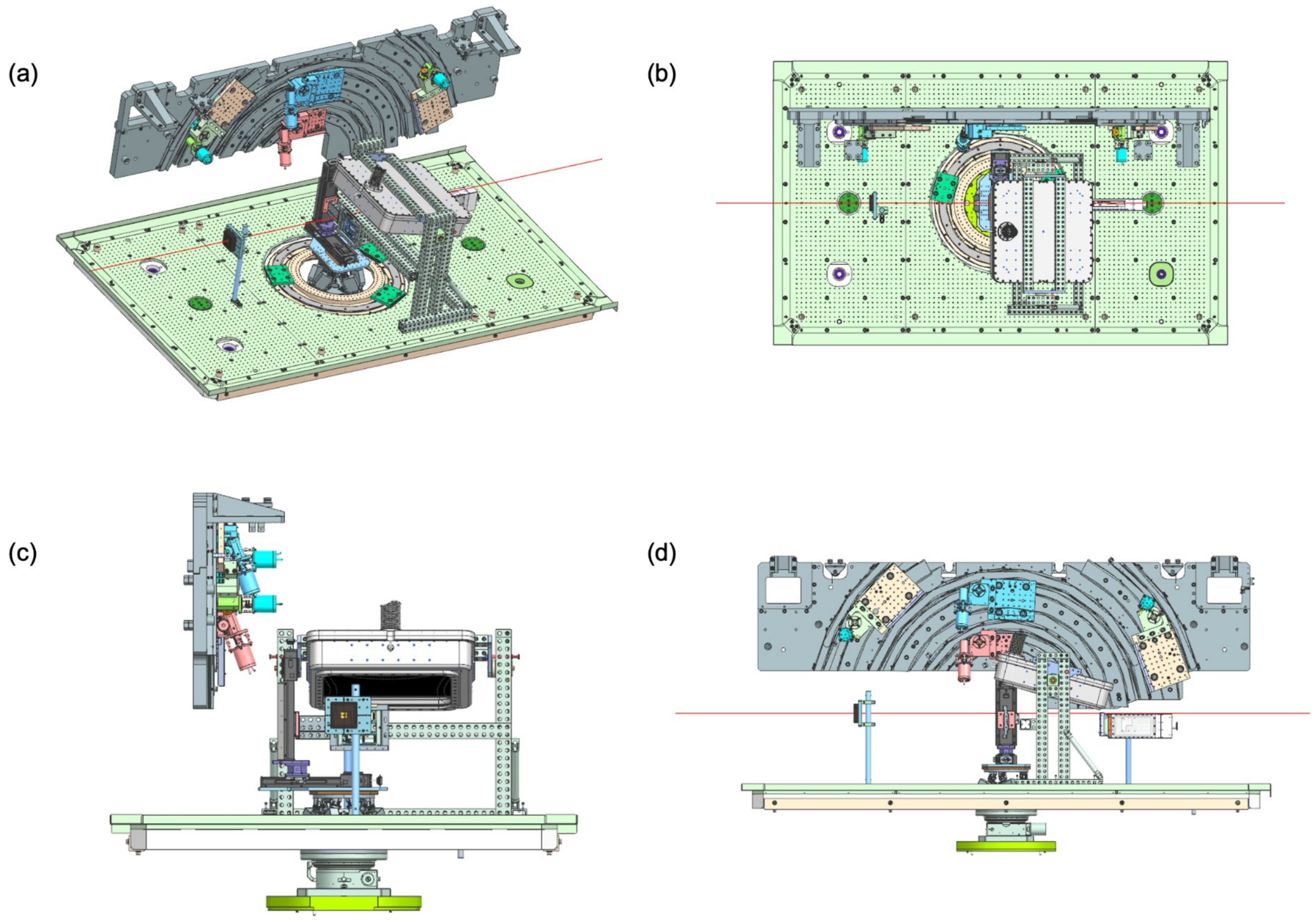

Figure S1 CAD drawings of the HED setup, with the path of the XFEL beam shown in red.

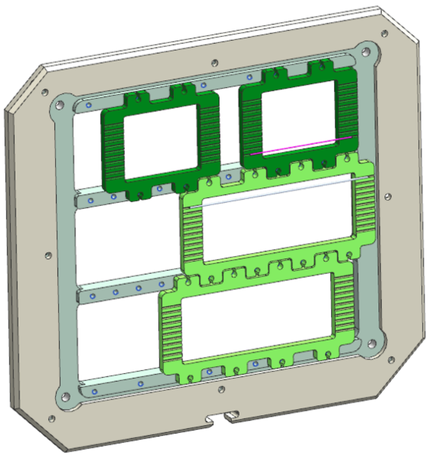

Figure S2 EUCALL sample holder with bespoke (green in the diagram) frames that can accommodate long and short capillaries of varying diameter. Flat plates can also be mounted (not shown).

Data Processing Corrections

Sensor Efficiency correction

The sensors of the Varex and Jungfrau are 0.55 mm thick CsI and 0.45 mm thick Si, respectively. We extracted the cosine of the angle of incidence from the calibrated pyFAI azimuthalIntegrator object directly and used this angle to calculate an effective detector thickness (d), which was combined with the theoretical absorption length (μ) to give a pixel-wise sensor efficiency correction.

Aluminium window transmission correction

We calculated the transmission of the aluminium windows based on the designed specifications and applied this to the detector images before azimuthal integration. Both detectors used a 0.4 mm-thick aluminium window. We extracted the cosine of the angle of incidence from the calibrated pyFAI azimuthalIntegrator object directly and inversely scaled the calculated $-\mu d$ by this array to give a pixel-wise window transmission correction. It is known that the Varex window does not remain flat after it is subject to the pressure differences in the HED interaction chambers (Gorman *et al.*, 2024). Our observation was that this mostly impacts the edges of the window, which, in our detector arrangement, made little difference to the data normalisation except perhaps at the very highest scattering angles. Hence, our assumption of a flat window was sufficient.

Detector Response

In the absence of a measured flat field from the supplier, we attempted to calculate a simple flat field correction to remove the most significant artefacts. The main features impacting our analyses were the "panelling" effect as seen in **Figure S3**. We then defined a flatfield correction consisting of 24 panels, each with a constant flat field value. We optimised the flat field by minimising the difference between neighbouring pixels across the panel boundaries.

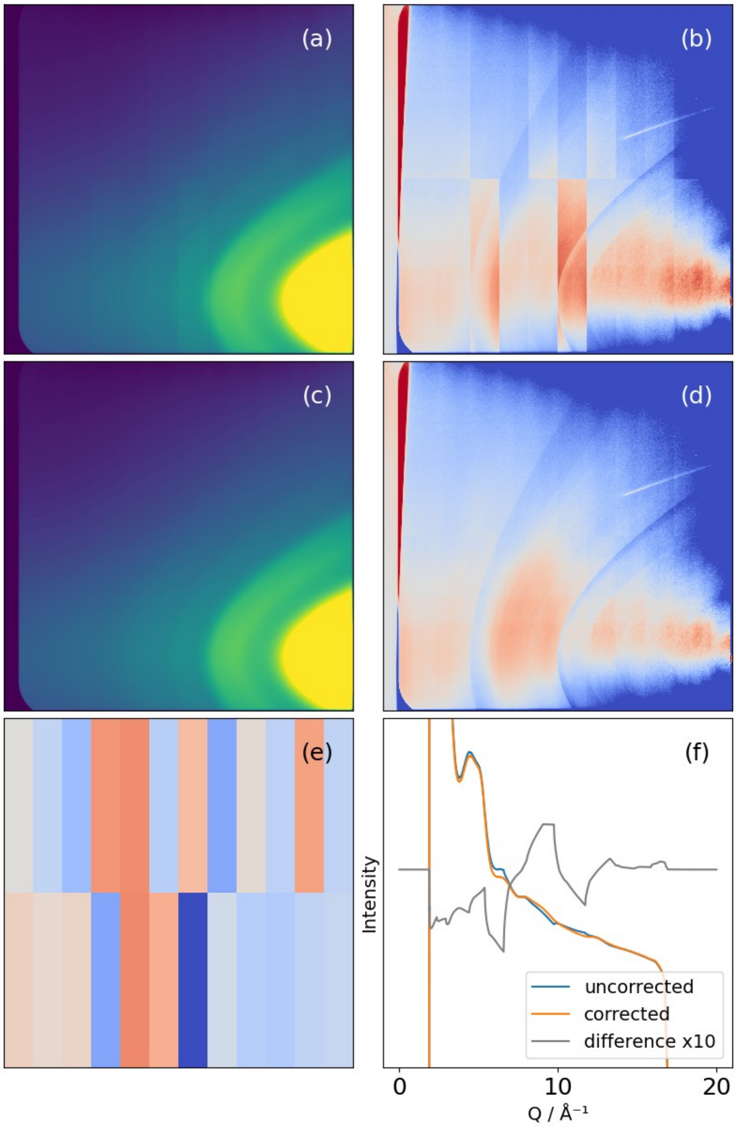

Figure S3 The flat field correction, showing the artefacts causing the largest issues and our resolution. **(a)** An exemplar dataset from the Varex detector, **(b)** the difference between these data and the back-calculated images calculated from the median filtered data, to emphasise the panelling, **(c)** the raw data with the flat field applied, **(d)** the difference between the flat field corrected data and the back-calculated images calculated from the median filtered data, to emphasise the panelling **(e)** the flat field, **(f)** the impact of the flat field on the azimuthally integrated data.

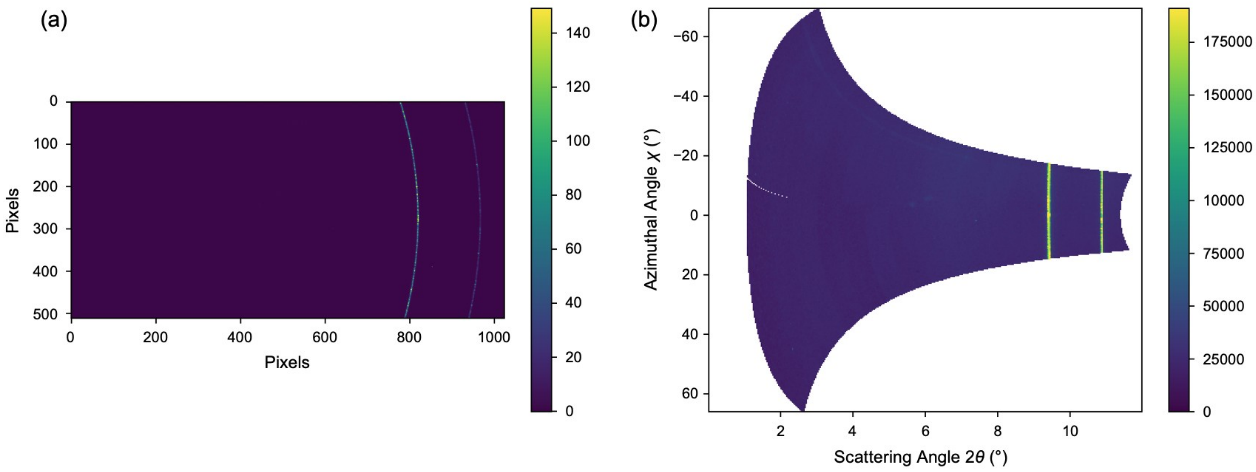

Figure S4 **(a)** Jungfrau detector image of a NIST CeO_2 674 powder. **(b)** Data plotted as a function of scattering and azimuthal angle showing vertical lines of intensity corresponding to the two highest *d*-spacing Bragg reflections in NIST CeO_2 674.

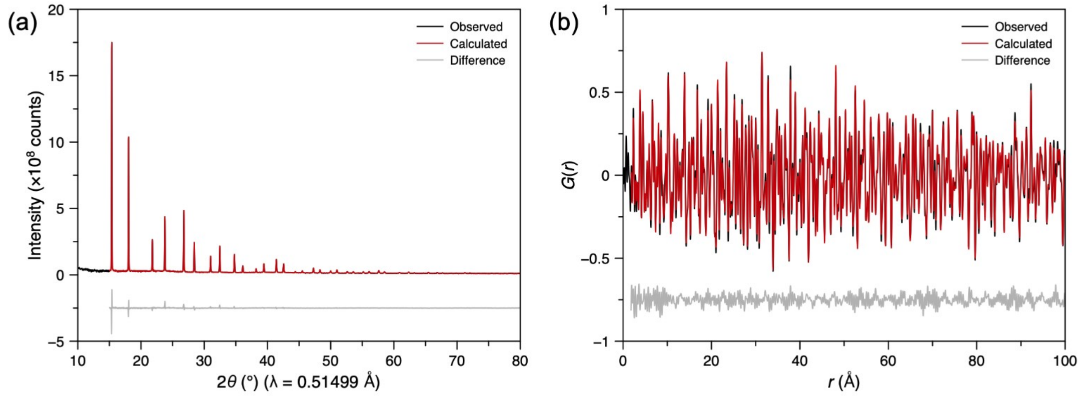

Figure S5 Si NIST 640b **(a)** Rietveld refinement against the powder diffraction pattern and **(b)** small-box refinement against the PDF. Each of the two datasets were used to refine the structural model independently.

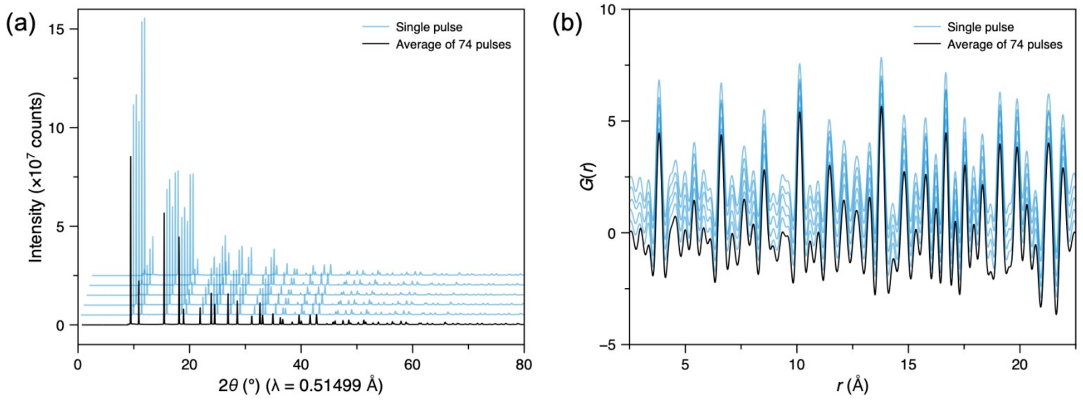

Figure S6 Representative single pulse data (blue lines) compared with the average data (black line) for NIST CeO_2 674. **(a)** Powder diffraction pattern (offset both horizontally and vertically for clarity) and **(b)** PDF (offset vertically).

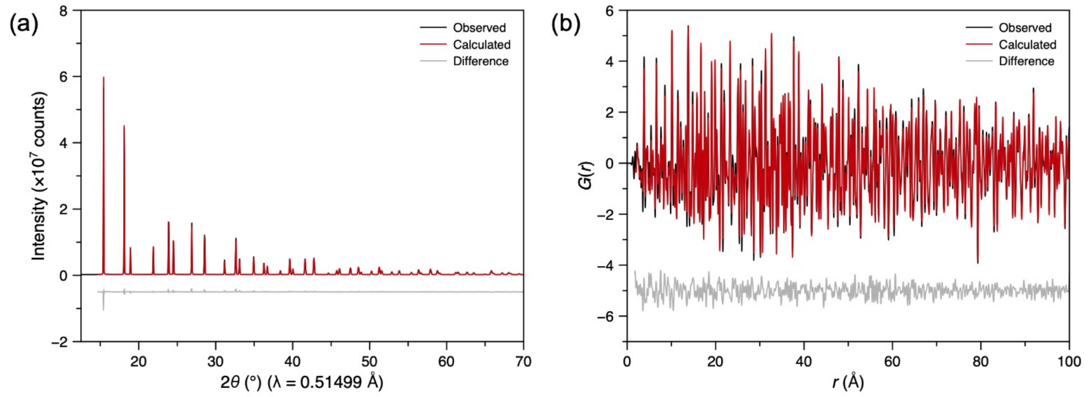

Figure S7 Refinements against the average data obtained for NIST CeO_2 674. **(a)** Rietveld refinement against the powder diffraction data and **(b)** small-box refinement against the PDF.

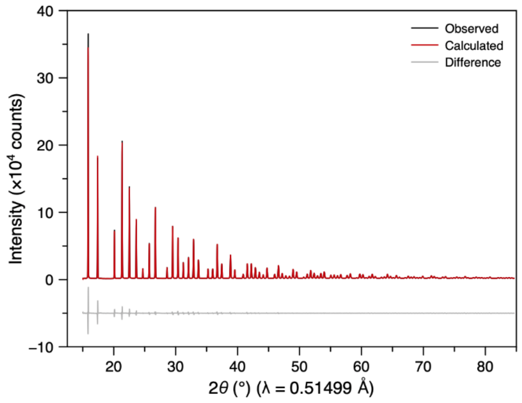

Figure S8 Rietveld refinement against the average NIST LaB_6 660b diffraction data.

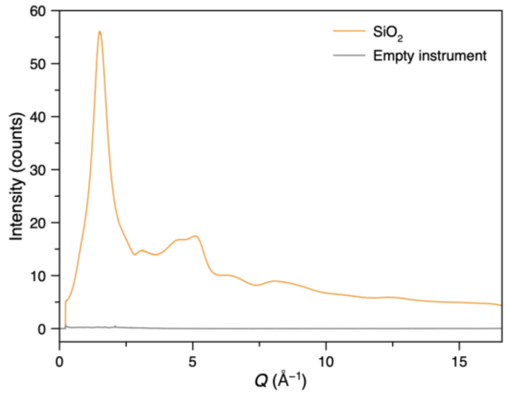

Figure S9 Comparison between the average SiO_2 diffraction data and data collected from the empty instrument.

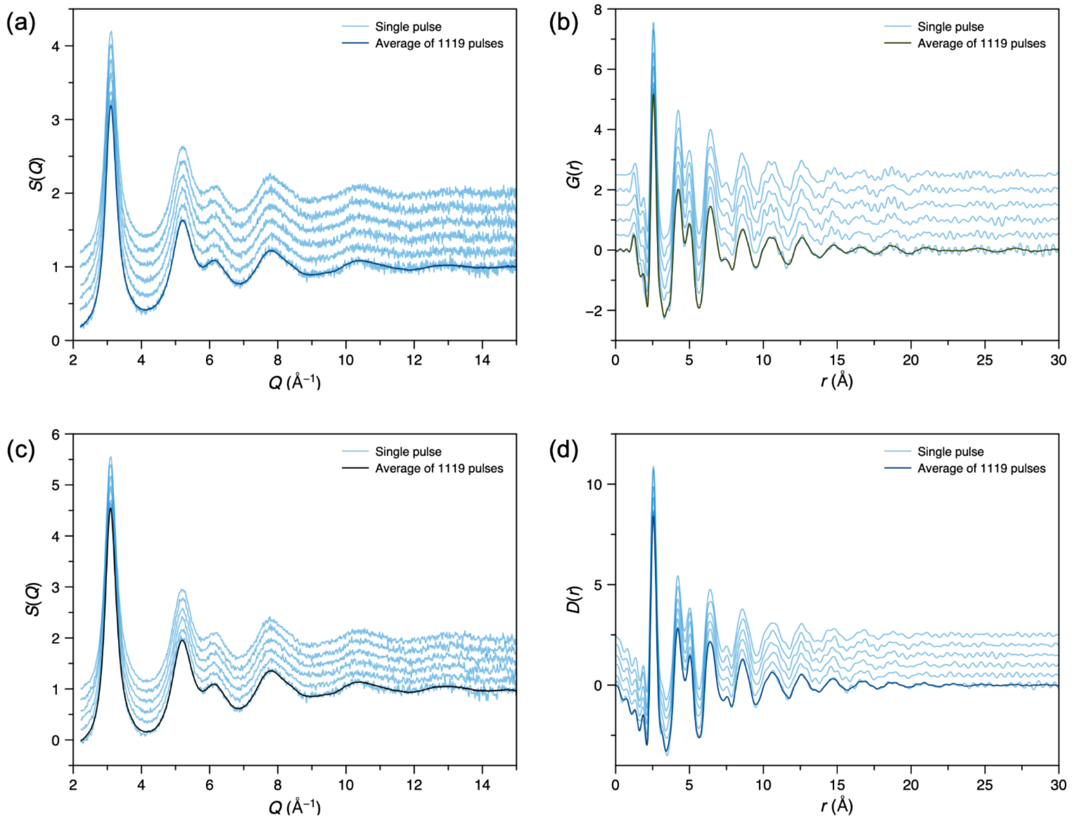

Figure S10 Representative examples of single pulse data (blue lines) obtained for the metallic glass, compared with the average data (black line). **(a)** and **(b)** show the $S(Q)$ and $G(r)$ obtained from PDFgetX3, respectively. **(c)** and **(d)** show the $S(Q)$ and $D(r)$ obtained from GudrunX, respectively. In (a) – (d), some data sets are offset vertically for clarity.

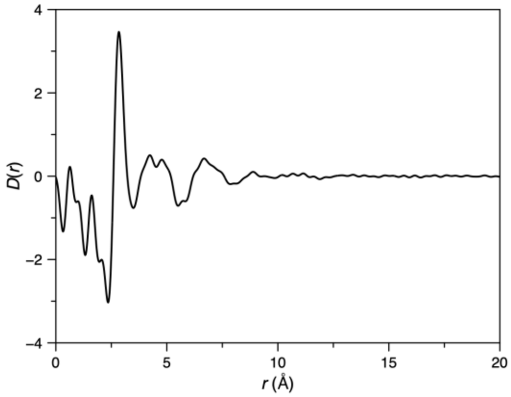

Figure S11 Average $D(r)$ obtained from water.

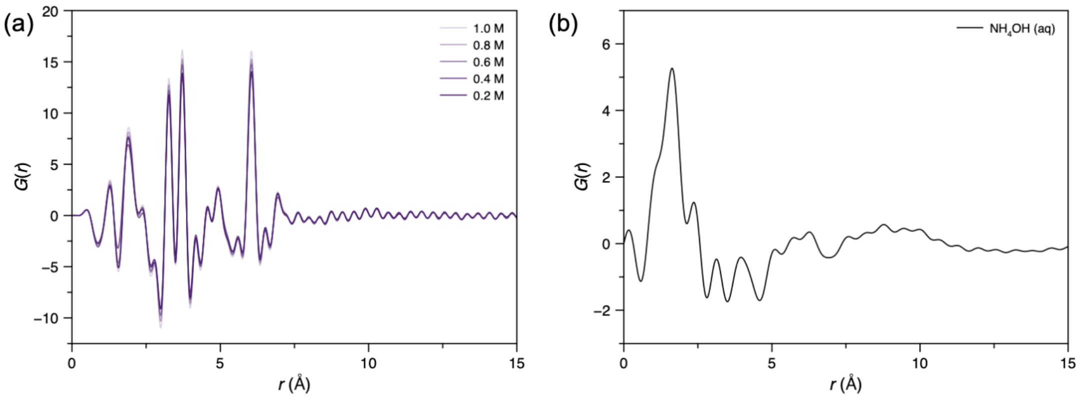

Figure S12 (a) Comparison between the difference PDFs of the Keggin cluster at various concentrations and **(b)** reference PDF of NH_4OH (aq), after background subtraction using a capillary loaded with water, measured at a synchrotron to illustrate the solvent-restructuring effects in solution.
